# Talent Identification in an English Premiership Rugby Union Academy: Multidisciplinary Characteristics of Selected and Non-selected Male Under-15 Players

**DOI:** 10.3389/fspor.2021.688143

**Published:** 2021-06-11

**Authors:** Francesco Dimundo, Matthew Cole, Richard C. Blagrove, Alexander B. T. McAuley, Kevin Till, Adam L. Kelly

**Affiliations:** ^1^Department of Sport and Exercise, Research Centre for Life and Sport Sciences, School of Health Sciences, Birmingham City University, Birmingham, United Kingdom; ^2^Worcester Warriors Rugby Football Club, Sixways Stadium, Worcester, United Kingdom; ^3^School of Sport, Exercise, and Health Sciences, Loughborough University, Loughborough, United Kingdom; ^4^Carnegie School of Sport, Leeds Beckett University, Leeds, United Kingdom

**Keywords:** talent development, Rugby Football Union, expertise, talent identification, selection, athlete development, LtAD

## Abstract

Entry into an academy can be a defining moment for a promising young player. The aim of this study was to explore the multidimensional characteristics that differentiated selected and non-selected male under-15 rugby union players at an English Premiership academy. Seventy-four players (mean age 14.6 ± 0.3 years: selected *n* = 29; non-selected *n* = 45) were measured across nine characteristics from four overarching factors: (a) anthropometric (*n* = 2), (b) physiological (*n* = 5), (c) cognitive (*n* = 1), and (d) birth quartile. An ANOVA compared differences between groups (selected vs. non-selected), whilst a Welch's *t*-test and Cohen's *d* were used for further comparisons. A multivariate logistic regression was also used to predict selection. Results showed significant differences between selected and non-selected players for anthropometric (*P* = 0.021) and physiological factors (*P* < 0.001). Moreover, relatively older players were overrepresented with 65% born in the first half of the year, whereas no significant differences were apparent for the cognitive test. More specifically, selected players possessed greater body mass (*P* = 0.022, *d* = 0.5) and handgrip strength (*P* = 0.020, *d* = 0.5) compared to non-selected players, whilst multivariate analysis showed the 20 m sprint explained 25.4% of the variance (*P* = 0.001). Overall, it appears selection into an English Premiership rugby union academy may be due to enhanced physical attributes rather than cognitive abilities.

## Introduction

Achieving professional status in sport is the quest of many young athletes across the globe (Till and Baker, 2020). Indeed, one of the increasing pressures for sport organizations is to identify promising young athletes and provide them with an optimal learning environment to facilitate long-term performance (Baker et al., 2013). The male rugby union (RU) talent pathway in England is comprised of an academy programme, delivered via 14 Regional Academies (currently aligned with 12 Premiership clubs, one Championship club, and one unaffiliated; Kelly et al., 2021b). Individuals are typically identified from age-grade or school rugby union, whereby they are selected at the end of the Under (U) 15 age group into Regional Academies (Till et al., 2020). Once selected into a Regional Academy at U15, the pathway consists of U18 and senior academy (e.g., U21) rosters to facilitate development toward the first team. Thus, it is plausible to suggest that initial selection into a RU academy at U15 can be a crucial moment for an aspiring young player.

Talent identification (TID) can be defined as recognizing young athletes with the potential to achieve expertise in a particular sport (Williams and Reilly, 2000). The TID process in RU is often influenced by a number characteristics, such as: (a) *anthropometric* (e.g., greater body size and mass; Fontana et al., 2015), (b) *physiological* (e.g., superior speed, strength, and power; Owen et al., 2020), (c) *cognitive* (e.g., advanced tactical skills; Sherwood et al., 2019), and (d) *birth quartile* (e.g., relatively older players overrepresented; Kelly et al., 2021a). From an anthropometric perspective, body mass and body fat percentage has been found to predict competition levels in youth RU players (Till et al., 2020; Dimundo et al., 2021). In a cross-sectional study on Italian players, Fontana et al. (2015) found that the lower the level of the player, the higher the percentage of fat mass was. Moreover, physiological attributes, such as sprint speed, strength, and power are regarded as important factors that differentiate between players based on age group, competition level, and position (Owen et al., 2020; Dimundo et al., 2021). For instance, Darrall-Jones et al. (2015) found that countermovement jump (CMJ) height, peak power, sprint momentum, acceleration speed, and isometric strength improved with age (i.e., U16–U21) in an English Premiership Regional Academy.

Cognitive characteristics, such as anticipation and decision-making skills (i.e., perceptual-cognitive expertise; see Mann et al., 2007 for a review), are also crucial for differentiating players based on ability levels, which has been explored in different rugby contexts including Australia, England, France, New Zealand and South Africa (Dimundo et al., 2021). As an example, Farrow et al. (2010) used video simulations to examine anticipatory skills, revealing that pattern recall could differentiate expert, intermediate, and novice Australian RU players. In addition, birth quartile appears to play an important role during initial selection into RU talent development pathways (Kelly et al., 2021a). Specifically, Kelly et al. (2021b) demonstrated that 42.5% of players selected into English Regional Academies at U15 across the last three seasons (2016–2019) were born in the first 3 months of the annual selection year (i.e., September, October, and November) compared to just 9.6% born in the last 3 months (i.e., June, July, and August). These phenomenon are commonly termed as relative age effects (RAEs; Cobley et al., 2009). Overall, since there are various factors that can influence selection into RU talent development pathways, it is important to consider a multidisciplinary research methodology whilst examining the TID process.

The initial selection into a RU academy at U15 is a critical time for all English Premiership clubs, since these players will form the core of the subsequent age groups for the proceeding years toward their respective first team. As part of forming the U16 age group, it is common practice for Regional Academies to hold an annual trial (or performance camp) for promising U15 players from their regional junior centers and developing player programme (Till et al., 2020). However, the multidisciplinary factors (i.e., anthropometric, physiological, cognitive, and birth quartile) that differentiate those who are selected, compared to those who are non-selected, are yet to be empirically evaluated. As such, the aim of this study was to explore the anthropometric, physiological, cognitive, and birth quartile characteristics that differentiated selected and non-selected U15 English Premiership RU academy players, as well as examine the factors that predicted selection. Moreover, a secondary aim of this study was to distinguish differences between selected and non-selected players based on position (i.e., forwards vs. backs).

## Methods

### Participants

Seventy-four participants (mean age 14.6 ± 0.3 years: selected *n* = 29; non-selected *n* = 45) from an English Premiership RU Regional Academy participated in this study. Participants were also divided by their preferred playing position (selected forwards *n* = 14; non-selected forwards *n* = 18; selected backs *n* = 15; non-selected backs *n* = 27) for further analysis. Ethical approval was granted by Birmingham City University via the Health, Education, and Life Sciences Research Ethics Committee.

### Procedures

The participants were invited to a four-day performance summer camp (i.e., annual trial) in an attempt to be selected for the U15 squad at an English Premiership RU Regional Academy. These participants are typically identified from community or school rugby, whereby the annual trial for the Regional Academy is delivered on an invitation-only basis (Till et al., 2020). Participants were pre-identified from existing Alongside specific RU training, participants were tested to record key performance parameters, which comprised of nine characteristics from the four overarching factors: (a) *anthropometric* (i.e., body height and mass), (b) *physiological* (i.e., 10 and 20 m sprint time, CMJ, isometric hip extension [IHE], and dominant handgrip strength), (c) *cognitive* (i.e., perceptual-cognitive video simulation test), and (d) *birth quartile* (i.e., date of birth). All physiological measures were collected following the same tests order, with a minimum of 3 min between tests, during day-1 of the performance camp. This approach allowed comparison between those who were subsequently selected and non-selected.

Participants' body height and mass were measured to the nearest 0.1 cm and 0.1 kg using a Seca Alpha stadiometer and calibrated Seca Alpha (model 220) scales wearing only shorts (e.g., Darrall-Jones et al., 2015). A standardized warm-up and two familiarization trials were performed before each physiological test. Sprint time over 10 and 20 m was recorded using timing gates (Brower Timing Systems, IR Emit. Draper, UT, USA). Each sprint started 30 cm behind the initial timing gate, with participants instructed to commence at a freely-chosen time and run maximally through the final 20 m timing gate (e.g., Darrall-Jones et al., 2015). A CMJ was performed with the participants hands placed on the hips while stood between two portable infrared recorders (Microgate, OptoGate, Italy) that recorded jump height to the nearest 0.1 cm. Participants were instructed to complete the CMJ starting from a standing position, moving to a self-selected depth (without overpassing the knees joint with their hip), and to jump as high as possible (e.g., Román et al., 2018). A portable back and leg dynamometer (Takei Scientific Instruments Co., Ltd, Niigata-City, Japan) was used to measure IHE. Participants stood on a portable platform and pulled a handle connected with the platform via a chain. They were required to maintain a standard straight knees, back, and flexed hip. Following familiarization, participants were instructed to pull as hard and fast as possible after a 3 s countdown for 5 s (Coldwells et al., 1994). Handgrip strength was measured using a handgrip dynamometer (Takei, 5401, Takei Scientific Instruments, Japan). Once an optimal position was determined by sitting and holding the tested hand's elbow 90° flexed, participants' were instructed to “squeeze” as hard as possible for a 5 s duration (Massy-Westropp et al., 2011) only using the preferred (strongest) hand. Strong verbal encouragement was provided during the maximal strength tests. Each test was completed three times with the best attempt recorded for analysis.

A perceptual-cognitive video simulation test was used to examine the participants' decision-making skill based on a combination of tactical situations, which have been shown to be valid and reliable measures for PCE research in several sport environments (e.g., Kelly et al., 2020). Fifteen video clips were carefully chosen from live rugby match footage, filmed from different elevated angles to provide a wide-range view of the pitch. Following a few seconds of general build-up play, the screen unexpectedly frozen for 8 s prior to a critical decision-making moment. At this point, a question with four possible options appeared on the frozen action and participants had to select an answer on their response sheet before the next clip automatically began. As per examination conditions, participants were seated separately for ~45 min and were unable to engage with each other. Participants overall score was ranked using percentiles (i.e., 90th; 75th; 50th; 25th; 10th) and then classified (i.e., 1 = excellent; 2 = good; 3 = average; 4 = low; 5 = poor) for analysis. The total accuracy of the participants' responses was recorded for analysis. Finally, each participant was assigned a birth quartile, which was calculated using their date of birth. The annual selection year was divided into four birth quartiles according to the English cut-off dates (birth quartile one [BQ1] = September, October, and November; BQ2 = December, January, and February; BQ3 = March, April, and May; BQ4 = June, July, and August; McCarthy and Collins, 2014).

These tests were designed as part of the 4 day performance summer camp in order to provide coaches and practitioners with objective data. Coaches and practitioners working for the Regional Academy were charged with selecting and deselecting the U15 players following the completion of the performance camp. These decisions are subjective in nature; although the objective data used as part of this study was provided to the coaches in order to facilitate their decisions. However, it is important to recognize that this data was not regularly collected by the Regional Academy prior to this study, thus coaches and practitioners may have a limited understanding in applying this data to their selection and deselection decisions.

### Statistical Analysis

Data were checked for normal distribution using a Shapiro–Wilk test. Scores were then normalized using *z*-scores [*z* = (*x*-μ)/δ], where *x* is the raw score, μ is the population mean, and δ is the population standard deviation. A multivariate analysis of variance (MANOVA) was used to calculate difference among the combined anthropometric and physiological factors both between selected and non-selected participants and positions, whereas a one-way analysis of variance (ANOVA) was used to explore the differences for the cognitive test. A Cohen's *d* was also used to calculate the effect size of these factors. Cohen's *d* effect size was calculated as reported in previous literature (Cohen, 1988) with threshold values of 0.20 (small), 0.50 (medium), 0.80 (large), with corresponding 95% confidence intervals (CIs). A Welch's *t*-test was then conducted for the eight variables from the anthropometric, physiological, and cognitive factors to compare selected and non-selected participants, as well as position-specific comparisons (i.e., forwards vs. backs).

For birth quartiles, a chi-square (χ^2^) goodness-of-fit was used to compare quartile distributions for selected participants against national norms (McHugh, 2013; Office for National Statistics, 2015). Since the χ^2^ does not reveal the magnitude of difference between quartile distributions, a Cramer's V was also used to report the effect size (0.00 and under 0.10, negligible; 0.10 and under 0.20, weak; 0.20 and under 0.40, moderate; 0.40 and under 0.60, relatively strong; 0.60 and under 0.80, strong; 0.80 and under 1.00, very strong; Ferguson, 2009). Finally, a binary logistic regression was performed to model selected and non-selected participants, which comprised of multivariate analysis performance test only for statistically significant variables evidenced in the Welch's *t*-test or χ^2^. The pseudo R-squared values, odds ratios (ORs), and 95% CIs were reported for each model. Significance was set for an α level of 0.05 with the statistical analysis conducted using IBM SPSS Statistics Version 24.

## Results

Results from the MANOVA and ANOVA showed that there was a significant difference between selected and non-selected players for both anthropometric (*P* = 0.021) and physiological (*P* < 0.001) characteristics. Further results from the Welch's *t*-tests revealed moderate to large differences between participants for body mass (selected = 69.9 ± 11.5 kg vs. non-selected = 63.5 ± 12.1 kg; *P* = 0.022, *d* = 0.53), handgrip strength (selected = 38.1 ± 7.2 kg vs. non-selected = 33.9 ± 8.0 kg; *P* = 0.020, *d* = 0.52), IHE (selected = 137.4 ± 22.6 kg vs. non-selected = 117.0 ± 23.8 kg; *P* < 0.001, *d* = 0.87), and 20 m sprint (selected = 3.33 ± 0.14 s vs. non-selected = 3.44 ± 0.21 s; *P* < 0.001, *d* = 0.75). When analysing groups based on position, selected forwards had greater IHE (144.3 ± 23.7 kg vs. 130.2 ± 12.6 kg; *P* = 0.054, *d* = 0.77) and 20 m sprint (3.40 ± 0.11 s vs. 3.53 ± 0.21 s; *P* = 0.041, *d* = 0.71) compared to non-selected forwards with large effect size differences. In comparison, selected backs had greater IHE (130.9 ± 20.2 kg vs. 108.3 ± 25.6 kg; *P* < 0.001, *d* = 0.95) and 20 m sprint (3.26 ± 0.13 s vs. 3.38 ± 0.18 s; *P* = 0.011, *d* = 0.78) compared to non-selected backs with large effect size differences. In addition, there was no significant differences between groups and positions for the perceptual-cognitive video simulation test. The descriptive statistics are reported in Table 1. The MANOVA for anthropometric and physiological factors and the ANOVA for cognitive factor are reported in Table 2. The Welch's *t*-test analysis is reported in Table 3.

**Table 1:** Descriptive statistics for selected and non-selected U15 players.

**Characteristic**	**Selected**			**Non-selected**		
	**Forwards (*n* = 14) mean ± SD**	**Backs (*n* = 15) mean ± SD**	**All players (*n* = 29) mean ± SD**	**Forwards (*n* = 18) mean ± SD**	**Backs (*n* = 27) mean ± SD**	**All players (*n* = 45) mean ± SD**
**Anthropometric**						
Body mass (kg)	77.2 ± 10.9	63.1 ± 7.2	69.9 ± 11.5	71.3 ± 9.6	58.3 ± 10.9	63.5 ± 12.1
Height (cm)	173.2 ± 3.6	170.5 ± 7.3	171.8 ± 5.9	175.1 ± 7.0	166.9 ± 10.1	170.2 ± 9.8
**Physiological**						
Handgrip (kg)	40.6 ± 7.3	35.8 ± 6.6	38.1 ± 7.2	36.8 ± 6.3	31.9 ± 8.5	33.9 ± 8.0
IHE (kg)	144.3 ± 23.7	130.9 ± 20.2	137.4 ± 22.6	130.2 ± 12.6	108.3 ± 25.6	117.0 ± 23.8
10 m sprint (s)	1.41 ± 0.05	1.34 ± 0.06	1.37 ± 0.06	1.45 ± 0.10	1.38 ± 0.09	1.41 ± 0.10
20 m sprint (s)	3.40 ± 0.11	3.26 ± 0.13	3.33 ± 0.14	3.53 ± 0.21	3.38 ± 0.18	3.44 ± 0.21
CMJ (cm)	28.8 ± 4.7	33.2 ± 5.8	31.1 ± 5.7	27.3 ± 5.3	31.5 ± 5.8	29.8 ± 5.9
**Cognitive**						
Perceptual-cognitive video simulation test (au)	2.9 ± 1.3	3.5 ± 1.1	3.2 ± 1.2	3.2 ± 1.5	3.3 ± 1.3	3.2 ± 1.3
**Birth Quartile**						
BQ	2 ± 1	3 ± 1	2 ± 1	2 ± 1	2 ± 1	2 ± 1

*Shows difference between selected and non-selected players and reports comparison among positions. SD, standard deviation; IHE, isometric hip extension; CMJ, countermovement jump; au, arbitrary unit; BQ, birth quartile*.

**Table 2:** MANOVA for the anthropometric and physiological factors and ANOVA for the cognitive factor.

**Factor**	**Selected vs. non-selected forwards (*P*)**	**Selected vs. non-selected backs (*P*)**	**All selected vs. all non-selected (*P*)**	**Selected forwards vs. selected backs (*P*)**
Anthropometric	0.031^*^	0.331	<0.021^*^	0.165
Physiological	0.246	0.020^*^	<0.001^*^	0.617
Cognitive	0.502	0.568	<0.989	0.453

*Significance set for P = 0.05*;

* *a statistical significance of ≤ 0.05*.

**Table 3:** *Z*-scores and Welch's *t*-tests for selected and non-selected players.

**Characteristic**	**Selected *z*-score (mean ± SD)**	**Non-selected *z*-score (mean ± SD)**	**Welch's *t*-test (*P*)**	**Cohen's *d***
**Body mass**				
Forwards	0.32 ± 1.04	−0.25 ± 0.92	0.124	0.58 (−0.14, 1.28)
Backs	0.31 ± 0.73	−0.17 ± 1.10	0.099	0.49 (−0.15, 1.13)
Selected vs. non-selected	0.31 ± 0.88	−0.20 ± 1.02	0.022^*^	0.53 (0.06, 1.01)
Selected forwards vs. backs			0.982	0.00 (−0.72, 0.73)
**Height**				
Forwards	−0.19 ± 0.62	0.15 ± 1.21	0.320	−0.33 (−1.03, 0.37)
Backs	0.25 ± 0.79	−0.14 ± 1.09	0.199	0.39 (−0.25, 1.03)
Selected vs. non-selected	0.04 ± 0.74	−0.02 ± 1.14	0.761	0.06 (−0.40, 0.53)
Selected forwards vs. backs			0.100	−0.61 (−1.35, 0.14)
**Handgrip**				
Forwards	0.31 ± 1.05	−0.23 ± 0.92	0.133	0.56 (−0.16, 1.27)
Backs	0.31 ± 0.82	−0.17 ± 1.06	0.111	0.49 (−0.16, 1.12)
Selected vs. non-selected	0.31 ± 0.92	−0.19 ± 1.00	0.020^*^	0.52 (0.05, 1.00)
Selected forwards vs. backs			0.999	0.00 (−0.73, 0.73)
**IHE**
Forwards	0.41 ± 1.23	−0.31 ± 0.65	0.054	0.77 (0.04, 1.49)
Backs	0.56 ± 0.78	−0.31 ± 0.99	<0.001^*^	0.95 (0.28, 1.60)
Selected vs. non-selected	0.49 ± 1.00	−0.31 ± 0.86	<0.001^*^	0.87 (0.38, 1.36)
Selected forwards vs. backs			0.707	−0.14 (−0.87, 0.59)
**10 m sprint**				
Forwards	−0.13 ± 0.32	0.10 ± 1.31	0.477	−0.23 (−0.93, 0.47)
Backs	−0.06 ± 0.44	0.03 ± 1.21	0.711	−0.09 (−0.73, 0.54)
Selected vs. non-selected	−0.09 ± 0.38	0.06 ± 1.24	0.432	−0.16 (−0.62, 0.31)
Selected forwards vs. backs			0.633	−0.18 (−0.91, 0.55)
**20 m sprint**				
Forwards	−0.38 ± 0.61	0.29 ± 1.15	0.041^*^	−0.71 (−1.42, 0.02)
Backs	−0.47 ± 0.73	0.26 ± 1.05	0.011^*^	−0.78 (−1.43, −0.12)
Selected vs. non-selected	−0.43 ± 0.66	0.27 ± 1.08	<0.001^*^	−0.75 (−1.23, −0.27)
Selected forwards vs. backs			0.709	0.14 (−0.59, 0.87)
**CMJ**
Forwards	0.17 ± 0.94	−0.12 ± 1.05	0.411	0.29 (−0.41, 0.99)
Backs	0.18 ± 0.99	−0.10 ± 1.01	0.381	0.28 (−0.35, 0.92)
Selected vs. non-selected	0.17 ± 0.95	−0.11 ± 1.01	0.222	0.29 (−0.18, 0.76)
Selected forwards vs. backs			0.965	−0.02 (−0.75, 0.71)
**Perceptual-cognitive video simulation test**				
Forwards	−0.14 ± 0.93	0.10 ± 1.07	0.499	−0.24 (−0.94, 0.46)
Backs	0.12 ± 0.90	−0.06 ± 1.06	0.546	0.19 (−0.45, 0.82)
Selected vs. non-selected	0.00 ± 0.91	0.00 ± 1.05	0.981	0.00 (−0.47, 0.46)
Selected forwards vs. backs			0.455	−0.28 (−1.01, 0.45)

*Shows difference between selected and non-selected players and reports comparison among positions. In the column headings indicate overall effects (significance set for P = 0.05). Post-hoc and Cohen's d effect size (90% confidence interval). IHE, isometric hip extension; CMJ, countermovement jump*;

* *a statistical significance of ≤ 0.05*.

Birth quartiles showed an higher proportion of those born in the first half of the year for selected participants (BQ1 = 28%, BQ2 = 38%, BQ3 = 10%, and BQ4 = 24%), although it was not statistically significant and had weak effect size [χ^2^(3) = 4.62, V = 0.28, *P* = 0.206]. Moreover, birth quartiles were significantly skewed for non-selected participants with a moderate effect size [χ^2^(3) = 9.34, V = 0.32, *P* = 0.025], whereby a higher proportion were born in the first half of the year (BQ1 = 38%, BQ2 = 29%, BQ3 = 27%, and BQ4 = 6%). With regards to position, both selected forwards [BQ1 = 36%, BQ2 = 36%, BQ3 = 14%, and BQ4 = 14%; χ^2^(3) = 2.59, V = 0.30, *P* = 0.458] and selected backs (BQ1 = 20%, BQ2 = 40%, BQ3 = 7%, and BQ4 = 33%; χ^2^ (3) = 3.99, V = 0.36, *P* = 0.262) birth quartile's were skewed toward the first half of the year with moderate effect sizes, although it was not statistically significant. Likewise, both non-selected forwards [BQ1 = 33%, BQ2 = 33%, BQ3 = 28%, and BQ4 = 6%; χ^2^(3) = 3.99, V = 0.32, *P* = 0.274] and non-selected backs [BQ1 = 41%, BQ2 = 26%, BQ3 = 26%, and BQ4 = 7%; χ^2^(3) = 5.96, V = 0.33, *P* = 0.113] birth quartile's were skewed toward the first half of the year with moderate differences, although it was not statistically significant. The birth quartile results are reported in Table 4.

**Table 4:** Birth quartile distributions by position vs. national norms.

**Cohort**	**BQ1 (*n*)**	**BQ2 (*n*)**	**BQ3 (*n*)**	**BQ4 (*n*)**	**Total (*n*)**	**X^**2**^**	**Cramer's V**	***P***	**Q1 vs. Q4**	**Q2 vs. Q4**	**Q3 vs. Q4**
	**%**	**%**	**%**	**%**	**%**	**(df = 3)**			**(OR, 95% CI)**	**(OR, 95% CI)**	**(OR, 95% CI)**
Selected forwards	5 (35.7%)	5 (35.7%)	2 (14.3%)	2 (14.3%)	14 (100%)	2.59	0.30	0.453	2.49 (0.27–22.55)	2.59 (0.28–23.70)	1.03 (0.08–12.02)
Selected backs	3 (20%)	6 (40%)	1 (6.7%)	5 (33.3%)	15 (100%)	3.99	0.36	0.267	0.59 (0.07–4.49)	1.24 (0.19–0.01)	0.20 (0.01–2.72)
Selected forwards and backs	8 (27.6%)	11 (37.9%)	3 (10.4%)	7 (24.1%)	29 (100%)	4.62	0.28	0.206	1.14 (0.27–4.81)	1.63 (0.40–6.62)	0.44 (0.08–2.41)
Non-selected forward	6 (33.3%)	6 (33.3%)	5 (27.8%)	1 (5.6%)	18 (100%)	3.88	0.32	0.274	5.99 (0.50–71.66)	6.23 (0.51–75.07)	5.15 (0.41–63.63)
Non-selected backs	11 (40.7%)	7 (25.9%)	7 (25.9%)	2 (7.5%)	27 (100%)	5.96	0.33	0.113	5.49 (0.87–34.60)	3.63 (0.54–24.31)	3.60 (0.54–24.10)
Non-selected forwards and backs	17 (37.8%)	13 (28.9%)	12 (26.7%)	3 (6.6%)	45 (100%)	9.34	0.32	0.025^*^	5.65 (1.29–24.74)	4.50 (1.00 −20.24)	4.12 (0.91–18.68)

*BQ1 = September, October, and November; BQ2 = December, January, and February; BQ3 = March, April, and May; BQ4 = June, July, and August. Birth quartile (BQ1–BQ4) distribution by positions, total number of players, and comparisons against national norm with odd ratio (OR) set at 95% of confidence interval (CI), significance set for P = 0.05*;

* *denotes a statistical significance of ≤ 0.05*.

The multivariate logistic regression model explained between 21% (Cox and Snell R square) and 29% (Nagelkerke R square) of the variance in selection (*P* = 0.001). Only the 20 m sprint made a statistically significant contribution to the model that predicted selection. In general, 20 m sprint time explained 25.4% of the variance (*r*^2^ = 0.254, *P* = 0.039). The multivariate logistic regression is reported in Table 5.

**Table 5:** Main variables for multivariate logistic regression for selection and positions.

**Cohort**	**Predictor**	**Coefficient β**	**SE**	**Wald's χ2**	**Odds Ratio (95% CI)**	**Log**	**Cox and**	**Nagelkerke**
						**likelihood**	**Snell *R*^**2**^**	***R*^**2**^**
**Forwards: selected vs. non-selected**						−18.87	0.198	0.265
	IHE	0.714	0.445	χ2(1) = 2578, *P* = 0.108	0.490 (−1.585; 0.158)			
	20 m sprint	−0.741	0.503	χ2(1) = 2174, *P* = 0.140	2.099 (−0.244; 1.727)			
	Constant	−0.319	0.405	χ2(1) = 0.620, *P* = 0.431	1.376 (−0.475; 1.113)			
**Backs: selected vs. non-selected**						−25.44	0.209	0.287
	IHE	0.884	0.499	χ2(1) = 3.131, *P* = 0.077	0.413 (−1.863; 0.095)			
	20 m sprint	−0.557	0.495	χ2(1) = 1264, *P* = 0.261	1.745 (−0.414; 1.528)			
	Constant	−0.820	0.400	χ2(1) = 4.200, *P* = 0.040	2.270 (0.036; 1.604)			
**All: selected vs. non-selected**						−40.74	0.212	0.287
	Body mass	0.378	0.370	χ2(1) = 1.042, *P* = 0.307	1.459 (0.706; 3.014)			
	Handgrip	0.282	0.391	χ2(1) = 0.522, *P* = 0.470	0.754 (0.351; 1.622)			
	IHE	0.661	0.399	χ2(1) = 2.741, *P* = 0.098	1.936 (0.886; 4.232)			
	20 m sprint	−0.805^*^	0.391	χ2(1) = 4.244, *P* = 0.039^*^	0.447 (0.208; 0.962)			
	Constant	−0.621	0.288	χ2(1) = 4.651, *P* = 0.031	1.861 (0.057; 1.186)			

*SE, standard error; IHE, isometric hip extension; 20 m sprint, sprinting time*;

* *a statistical significance of ≤ 0.05*.

## Discussion

Key findings suggest that those who were selected into the Regional Academy were significantly heavier, stronger, and faster over 20 m compared to their non-selected peers, with effect sizes for anthropometric, physiological, and cognitive factors ranging from small to large. Further multivariate logistic regression also revealed that only the 20 m sprint was a significant predictor for selection; irrespective of playing position. With regards to birth quartile and the cognitive factor, there was no statistically significant differences reported for selected players, despite being overrepresented in the first two birth quartiles (i.e., BQ1 = 28% and BQ2 = 38%) compared to the second two birth quartiles (i.e., BQ3 = 10% and BQ4 = 24%).

When comparing anthropometric characteristics findings (i.e., body height and mass) with other selected RU players, some similarities and variations occur based on other studies across other nationality. As an example, Nutton et al. (2012) reported similar body mass in Scottish U15 RU players (175.0 ± 7.0 cm; 68.0 ± 11.4 kg), although they appeared to be considerably taller. The population of the present study was also shorter (171.8 ± 5.9 cm), as well as lighter (69.9 ± 11.5 kg), than South African U15 RU players (175.0 ± 6.0 cm, 75.9 ± 13.2 kg; Grobler et al., 2017). Whereas, they were taller and heavier when compared to their Brazilian U15 RU equivalents (169.7 ± 12.1 cm, 63.8 ± 10.9 kg; Kobal et al., 2016). In a recent systematic review by Owen et al. (2020), it was reported that body height and mass in U15 RU players ranged from 169.7 to 175.0 cm and 63.8 to 75.9 kg, respectively; which is in line with the present findings. Thus, it is important to consider national youth sport culture (e.g., individual talent pathways, sport popularity, and national population) during the TID process, since variations in anthropometric characteristics can be considerable (see Dimundo et al., 2021 for a review).

When analysing anthropometric data by position, both selected forwards (173.2 ± 3.6 cm, 77.2 ± 10.9 kg) and selected backs (170.5 ± 7.3 cm, 63.1 ± 7.2 kg) presented similar characteristics to those reported in a French U15 academy (forwards = 175.9 ± 7.0 cm, 72.5 ± 9.8 kg; backs = 169.5 ± 6.5 cm and 60.8 ± 8.2 kg; Sedeaud et al., 2013). Indeed, body mass was pivotal when distinguishing selected RU players (Dimundo et al., 2021) in both a South African academy (Pienaar et al., 1998) and in New Zealand at senior international level (Quarrie et al., 1996). Moreover, similar to the present study's findings, Barr et al. (2014) showed that body mass, but not height, differentiated U20 and international RU players. The variation in anthropometric measures among playing positions, although not statistically significant, align with the understanding that forwards and backs require diverse physical characteristics to perform key roles and cope with position-specific demands of the game (Owen et al., 2020). Together, these results demonstrate that anthropometric characteristics, and in particular body mass, appear to be an important factor to consider during TID in U15 RU players. However, although not verified in this current study, it is important to recognize that, as reported in a recent systematic review (Dimundo et al., 2021), body mass depends on multiple factors (e.g., fat mass, lean mass, bone mass, and water), whilst higher-level players usually possess lower fat mass than lean mass. As such, these findings offer an important benchmark for coaches and practitioners when selecting U15 RU players, as well as highlighting the differences between positions.

Strength parameters have been shown to differentiate by age, competition levels, and position of young RU players across various environments (e.g., Pienaar et al., 1998; van Gent and Spamer, 2005; Spamer and De la Port, 2006; Hansen et al., 2011; Grobler et al., 2017; Owen et al., 2020). In this current study, selected players reported superior handgrip strength when compared to non-selected players. Indeed, similar conclusions have been reported in Portuguese (Vaz et al., 2019) and Scottish (Nutton et al., 2012) RU academy players, whereby it was suggested that handgrip strength should be one of the measures included in a battery of tests during the TID process since it was deemed a practical, safe, reliable, and valid method to detect a standard measure of strength in youths. Assessing force generating characteristics during the isomeric pull in RU has also been considered as a safe and useful tool to monitor progress across RU academies, since the technical requirement for these tests are less demanding compared to other traditional whole body strength tests (Darrall-Jones et al., 2015; Owen et al., 2020). Although not significant in multivariate logistic regression, all selected players in this study possessed greater handgrip and IHE measures than non-selected players, and selected forwards outperformed selected backs. However, it was not surprising that forwards possessed higher force than backs, since their playing-position requires them to produce higher isometric force during a game (Quarrie and Wilson, 2000). These results are in agreement with recent findings in RU (Owen et al., 2020), and demonstrate that whole body strength is an important factor to consider when approaching TID in RU due to its application in a multitude of key actions required in this contact sport (Till et al., 2020). Although results of this current study could have been influenced by an overrepresentation of relatively older participants and by the analysis of other characteristics of strength measures (i.e., relative strength), it also reveals how position-specific factors are already being influenced by physiological characteristics during initial entry into an academy at U15.

Sprint speed has been considered an important physiological quality in RU since it is associated with a range of performance outcomes, such as distance covered, evasion, and line and tackle breaks (Smart et al., 2014). It has been also used as one method to predict future talent in an Italian U16 RU academy (Fontana et al., 2017), indicating that it is worth monitoring this characteristic for optimal TID. In the current investigation, selected players possessed superior 20 m sprint times compared to non-selected players. Importantly, the 20 m sprint was the only predictive characteristic of selection in the current cohort. More specifically, those who possessed a faster 20 m sprint were up to 1.4 times more likely to be selected. A possible explanation for the importance of sprint speed in RU is that greater sprint characteristics have typically been correlated with greater momentum, which is believed to be fundamental in RU (Darrall-Jones et al., 2016; Jones et al., 2018). Thus, although momentum was not considered in this research, it is not surprising that fastest players were selected in the current academy squad. In addition, the present investigation found speed differences among playing positions. For instance, although 20 m sprint speed was an important factor for all players to possess, this was position-dependent whereby backs were generally faster than forwards. Therefore, in agreement to Jones et al.'s (2018) findings, 20 m sprint time can be considered one of the most valuable measures to include in a battery of tests when coaches aim to optimize TID during selection into their U15 cohort.

Cognitive skills are important factors to consider when selecting athletes in different sports (Mann et al., 2007). Although previous research in RU has suggested that superior cognitive skills differentiate playing levels (e.g., Farrow et al., 2010; Chiwaridzo et al., 2019a,b; den Hollander et al., 2019; Chiwaridzo et al., 2020; Runswick et al., 2020), results from this study did not report any statistical difference between selected and non-selected players. The outcomes of the present investigation could be justified by the fact that perceptual-cognitive qualities in U15 RU players may not have peaked at this stage of development. As an example, players may not have accumulated an adequate volume of hours in practice activities to develop athlete functionality at this entry level (Rothwell et al., 2020). Another explanation for this outcome could be due to the fact that coaches may have been focused on (and perhaps biased by) anthropometrical and physiological characteristics possessed by participants. In contrast, however, present findings on positional differences align with those of Runswick et al. (2020), whereby no statistical differences were reported in anticipation skills between forwards and backs. In summary, perceptual-cognitive skills remain an inconclusive measure for selection into a RU academy. Further study is encouraged to explore the implications of perceptual-cognitive skills on selection into RU academies, as well as incorporating a range of technical and psychosocial characteristics in holistic TID research methodologies.

Based on the common prevalence of RAEs in male RU, it was not surprising that there was an overrepresentation of selected players born between September and February in this current investigation (although this was only statistically significant for non-selected players). Specifically, the birth distribution revealed that almost twice as many players were selected from the first half of the year (*n* = 19; 66%) when compared to the second half of the year (*n* = 10; 34%). Non-selected players were significantly more likely to be born in the first half of the year (*n* = 30; 67%) compared to the second half of the year (*n* = 15; 33%). The percentages obtained reflect those reported in U7–U19 Welsh recreational RU clubs (BQ1 = 29% vs. BQ4 = 22%; Lewis et al., 2015), U13–U16 English regional representative squads (BQ1 = 38% vs. BQ4 = 10%; Roberts and Fairclough, 2012), English Regional Academies (BQ1 = 42% vs. BQ4 = 8%; McCarthy and Collins, 2014), and senior international levels (BQ1 = 32% vs. BQ4 = 20%; Kearney, 2017). Together, these results suggest that early born players may have an advantage over later born athletes during the initial phase of the TID process, since both selected and non-selected players are overrepresented. To be specific, the entry point into the Regional Academy appears to be biased toward their invitations to attend the performance camp; regardless of subsequent (un)successful selection (BQ1 and BQ2 = 66.2%). Moreover, the selection of relatively older players may be due to the fact that older players are likely to be more mature than younger ones (see Cobley et al., 2009). In U15 players, this may have resulted in relatively older players being faster and stronger than their relatively younger counterparts due to being less-developed. As such, Regional Academies are encouraged to explore alternative approaches to athlete selection (e.g., age-ordered shirt numbering; selection quotas; avoiding early deselection; flexible chronological approach) and group banding policies (e.g., age and anthropometric bands; bio-banding; playing-up and playing-down; see Webdale et al., 2020 for a review). Indeed, these could offer useful evidence-based guidelines in the future for other organizations and coaches to adopt practical solutions to RAEs as part of their TID procedures.

### Limitations and Future Directions

There are often methodological challenges when researching high-performance youth populations. In the context of this current study, although a relatively large representative sample of participants were examined (i.e., one of only fourteen Regional Academies across the country), the sub-analysis for position-specific study (i.e., forwards and backs) could have been influenced due to the sample size. It is also important to recognize that this study is susceptible to the individual academy's approach to TID, thus this sample may not be representative of all Regional Academy selection decisions. There may also be the case that the assessment of perceptual-cognitive skills in this current study could have been influenced by its sensitivity, possibly influencing the final results. Body mass was only recorded in kg and there was not the possibility to detect other important information such as fat and lean mass due to time constraints. Moreover, as the maturation status of the players was not assessed, it is not known to what extent this impacted selection. In addition, this study did not include the measurement of other important characteristics previously shown to be significant predictors of performance and selection (i.e., technical and psychosocial; Dimundo et al., 2021). Authors did not have information regarding participants' previous playing experience, which could have provided a better context of the population. The present findings may help selectors to refine their TID process, however, their selection reflects both their subjective perspectives and the estimated players' potential. Finally, it is also important to note that this study is cross-sectional in design, as such it does not take into account the dynamic, longitudinal nature of athlete development.

Future research is encouraged to include a more holistic and longitudinal protocol when assessing Regional Academy selection. As an example, longitudinal investigations should consider collecting the examined variables from a wider population by (a) including other performance factors (i.e., technical and psychosocial), and (b) expand performance indicators (i.e., including additional factors such as momentum), to study the ecological dynamic characteristics of the TID process (Till et al., 2013, 2015). The complex nature of the TID process is multitudinous by nature. Thus, selectors should act with caution when interpreting these outcomes, and are recommended not to base their selections *solely* on anthropometric and physiological qualities, and instead use these objective measures to complement their performance camps and decision-making processes on selection. Moreover, literature regarding athlete development suggests that due to greater physical characteristics being associated with early development, coaches should consider benchmarks based on biological age rather than chronological age (Malina et al., 2019; Kelly et al., 2021b). Lastly, as reported by Huijgen et al. (2014), coaches cognitive bias should be taken into account when examining players on physiological and technical variables for future researches in TID.

## Conclusion

This is the first study that has incorporated a multidisciplinary research design to compare selected and non-selected U15 RU players at an English Premiership Regional Academy. It appears anthropometric and physiological qualities are more predictive of selection when compared to cognitive characteristics and birth quartiles. Specifically, it is suggested that body mass, strength, and speed are part of a battery of tests that formulate part of the TID process during selection into Regional Academies. Moreover, Position-specific differences should also be considered also during early stages of TID. In addition, although birth quartile distribution was only statistically significant in the non-selected cohort, coaches and practitioners employed in youth RU should consider this as part of a holistic selection framework so potential talent is not missed. Future research is encouraged to adopt a multidimensional and longitudinal approach when investigating TID in RU, to build on this current study and better understand the selection processes in Regional Academies.

## Data Availability Statement

The raw data supporting the conclusions of this article will be made available by the authors, without undue reservation.

## Ethics Statement

The studies involving human participants were reviewed and approved by Birmingham City University via the Health, Education, and Life Sciences Research Ethics Committee. Written informed consent to participate in this study was provided by the participants' legal guardian/next of kin.

## Author Contributions

FD and AK contributed to conception and design of the study. FD organized the database and performed the statistical analysis. FD, AM, and AK contributed to the first draft of the manuscript. All authors contributed to manuscript revisions and approved the submitted version.

## Conflict of Interest

The authors declare that the research was conducted in the absence of any commercial or financial relationships that could be construed as a potential conflict of interest.
